# Personality and Gastric Cancer Screening Attendance:  A Cross-Sectional Analysis from the Miyagi Cohort Study

**DOI:** 10.2188/jea.JE20080024

**Published:** 2009-01-30

**Authors:** Shizuha Arai, Naoki Nakaya, Masako Kakizaki, Kaori Ohmori-Matsuda, Taichi Shimazu, Shinichi Kuriyama, Akira Fukao, Ichiro Tsuji

**Affiliations:** 1 Division of Epidemiology, Department of Public Health and Forensic Medicine, Tohoku University Graduate School of Medicine, Sendai, Japan; 2 Division of Behavioral Medicine, Department of Functional Medical Science, Tohoku University Graduate School of Medicine, Sendai, Japan; 3 Department of Public Health, Yamagata University Graduate School of Medicine, Yamagata, Japan

**Keywords:** attendance, cross-sectional study, gastric cancer screening, Japanese, personality

## Abstract

**Objective:**

To determine the associations between personality subscales and attendance at gastric cancer screenings in Japan.

**Methods:**

A total of 21,911 residents in rural Japan who completed a short form of the Eysenck Personality Questionnaire-Revised (EPQ-R) and a questionnaire on various health habits including the number of gastric cancer screenings attended were included in the primary analysis. We defined gastric cancer screening compliance as attendance at gastric cancer screening every year for the previous 5 years; all other patterns of attendance were defined as non-compliance. We defined gastric cancer screening visiting as attendance at 1 or more screenings during the previous 5 years; lack of attendance was defined as non-visiting. We used logistic regression to estimate the odds ratios (ORs) of gastric cancer screening compliance and visiting according to 4 score levels that corresponded to the 4 EPQ-R subscales (extraversion, neuroticism, psychoticism, and lie).

**Result:**

Extraversion had a significant linear, positive association with both compliance and visiting (trend, *P* < 0.001 for both). Neuroticism had a significant linear, inverse association with compliance (trend, *P* = 0.047), but not with visiting (trend, *P* = 0.21). Psychoticism had a significant linear, inverse association with both compliance and visiting (trend, *P* < 0.001 for both). Lie had no association with either compliance or visiting.

**Conclusion:**

The personality traits of extraversion, neuroticism, and psychoticism were significantly associated with gastric cancer screening attendance. A better understanding of the association between personality and attendance could lead to the establishment of effective campaigns to motivate people to attend cancer screenings.

## INTRODUCTION

Japan has one of the highest gastric cancer mortality rates in the world. According to World Health Organization statistics from 2002, the gastric cancer mortality rate in Japan (39.9 per 100,000) was among the highest in the Organization for Economic Co-operation and Development (OECD) countries (eg, Republic of Korea = 30.9; Portugal = 29.4; Hungary = 20.4; U.K. = 12.6; USA = 4.7).^1^

To control gastric cancer mortality in Japan, physicians and health care providers have conducted gastric cancer screenings since the 1960s. The effectiveness of gastric cancer screening in decreasing gastric cancer mortality among participants has been proven in a number of case-control^2^^–^^5^ and cohort studies.^6^^,^^7^ With subsidies from national and local governments, gastric cancer screenings are widely available throughout Japan.

According to the National Survey, however, the rate of participation in gastric cancer screening in 2004 was only 22%.^8^ Although this figure was higher than those for breast cancer screening (19%) and colorectal cancer screening (18%), measures to increase participation are urgently required.

Numerous factors influence the participation rate, including age, sex, marital status, education, annual income, past medical history, family history of cancer, the out-of-pocket charge for the screening test, and health-related lifestyle factors such as smoking.^9^^–^^11^ Psychological state is also considered to be one such factor. Past studies indicated that anxiety and depression were associated with cancer screening attendance. Two studies have noted that anxiety and depression scores were lower in those who attended cancer screening programs than in those who did not.^11^^,^^12^ Personality is another factor that might influence attendance. Breast cancer screening attendees tended to have a higher score in extraversion,^13^ and a lower score in neuroticism,^14^ than non-attendees.^13^^,^^14^ However, one study showed no association between personality and gastric cancer screening attendance.^15^

It should be noted that previous studies were limited by small sample size [147^12^–756^14^] and insufficient control for potential confounders such as socioeconomic status,^11^^,^^12^^,^^15^ past history of disease,^11^^–^^13^^,^^15^ and family history of cancer.^12^^,^^13^^,^^15^ Moreover, these studies were conducted mainly in Western countries,^11^^–^^14^ and it is uncertain whether their findings apply to other countries, in which the system for cancer screening and individuals’ attitudes to cancer screening differ. Better understanding of the association between personality and attendance at cancer screenings could lead to an effective campaign to motivate people to attend cancer screening programs.

The purpose of the present study was to examine the association between personality and gastric cancer screening attendance in a population-based sample. We chose gastric cancer screening because the rate of participation in gastric cancer screening in the past year was high in this study population (men 59.3%, women 56.8% in 1990).^7^ As compared with previous studies,^11^^–^^15^ the present study has the largest number of subjects and the most comprehensive set of covariates for multivariate adjustment.

## METHODS

### Study population

We have reported the design of this Miyagi Cohort Study in detail elsewhere.^16^ Briefly, from June through August 1990 we delivered 2 self-administered questionnaires to all 51,921 residents aged 40–64 years in 14 municipalities of Miyagi Prefecture—in rural, northern Japan. The first questionnaire inquired about various health habits, and the second was the Japanese version of the short form of the Eysenck Personality Questionnaire-Revised (EPQ-R).^17^ The questionnaires were delivered to, and collected at, the participants’ residences by members of health promotion committees appointed by the municipal governments. The response rate for the first questionnaire was 91.7% (n = 47,605), and 79.7% (n = 41,424) of the respondents to the first questionnaire responded to the second questionnaire. The study protocol was approved by the institutional review board of the Tohoku University Graduate School of Medicine. We considered the return of self-administered questionnaires signed by the subjects as consent to participate in the study.

### Gastric cancer screening in Japan

In Japan, a gastric cancer screening program has been in existence since 1960. It is available for all residents of Japan aged 40 years and over. Local governments provide residents with an opportunity for gastric cancer screening every year. The standard method for gastric cancer screening is the upper GI series using barium. The out-of-pocket charge for the examinee is free or low, at most 1000–2000 yen (10–20 U.S. dollars). If positive results are detected at screening, the individual is recommended to undergo diagnostic workup, which is covered by national health insurance.^18^

### Eysenck personality questionnaire-revised (EPQ-R)

The second questionnaire was a Japanese translation of the original English version of the EPQ-R Short Form, one of a series of personality inventories developed by Eysenck and colleagues.^19^ The EPQ-R has 48 questions with dichotomized responses (yes or no); there are 12 questions for each of 4 personality subscales (extraversion, neuroticism, psychoticism, and lie). Scores on each subscale range from 0 to 12, with higher scores indicating a greater tendency to possess the personality trait represented by each subscale. Extraversion represents sociability, liveliness, and surgency; neuroticism represents emotional instability and anxiousness; psychoticism represents tough-mindedness, aggressiveness, coldness, and egocentricity; and lie represents unsophisticated dissimulation and social naivety or conformity.^20^

Hosokawa et al, who developed the Japanese version of the EPQ-R, examined its reproducibility and validity among 329 college students and 253 adults. Cronbach’s *α* coefficient was greater than 0.70 for all subscales except psychoticism. Test–retest reliability coefficients for the 4 subscales over a 6-month period ranged from 0.70–0.85, indicating substantial stability. Confirmatory factor analysis supported the original theoretical structure of the 4 scales proposed by Eysenck and colleagues. Scores on the 4 subscales were highly correlated with scores on similar subscales in the Japanese versions of the Sixteen Personality Factor Questionnaire^21^ and the Maudsley Personality Inventory,^22^ indicating that the questionnaire had a high degree of concurrent validity.^17^

### Gastric cancer screening attendance

The first questionnaire asked, “How many times did you participate in gastric cancer screening during the last 5 years?” The participants were asked to provide the number of attendances.

We examined the association between personality and attendance at gastric cancer screening by using 2 different definitions: compliance and visiting. We defined attendance at gastric cancer screening every year for 5 years as gastric cancer screening compliance; all other attendance patterns were defined as non-compliance. We defined attending at least one screening during the last 5 years as gastric cancer screening visiting; complete lack of attendance was defined as gastric cancer non-visiting.

### Demographic variables and health habits

The first questionnaire inquired about demographic variables; self-reported height and weight; personal and family histories of cancer and other diseases; health habits including smoking, alcohol consumption, and diet; use of health services; marital status; and education, as well as cancer screening attendance.

### Statistical analyses

Of the 41,424 participants who responded to the 2 questionnaires, we excluded 54 participants who answered only “yes” or only “no” to all 48 items and 8600 participants for whom responses to any of the 48 items in the EPQ-R were missing. We further excluded 730 participants who had had cancer diagnosed at the time of the baseline survey. We also excluded 2437 participants who indicated that the 2 questionnaires had been completed with the aid of other family members, because we believed that such aid might have affected the response patterns of the study participants. We further excluded 4521 participants who reported a history of peptic ulcer, because of the effect this disorder may have had on gastric cancer screening attendance. We also excluded 3171 participants who did not answer the question about gastric cancer screening attendance. Consequently, 21,911 participants (9839 men and 12,072 women) remained for the analysis.

Each personality subscale was divided into 4 categories to obtain approximately equal quartiles. We used multivariate unconditional logistic regression to estimate odds ratios (ORs) for gastric cancer screening compliance and gastric cancer screening visiting for each category of personality subscales, with the lowest category treated as the reference group. Trend tests were performed by treating personality subscales as continuous variables.

In these analyses, we regarded the following data as covariates: age (continuous variables); sex; body-mass index (BMI) in kg/m^2^ (<18.5, 18.5–24.9, ≥25.0); family history of cancer (presence or absence); histories of diseases including stroke, hypertension, myocardial infarction, renal diseases, liver diseases, gallstone diseases, diabetes mellitus, and tuberculosis (presence or absence); time spent walking in hours per day (≤0.5, 0.5–1.0, ≥1.0); smoking status (current smoker, ex-smoker, never smoker); alcohol consumption (current drinker, ex-drinker, never drinker); marital status (married, separate/divorced/widowed, single); education (in school until age ≤15, 16–18, or ≥19 years).

In addition to sex and age, we included the following variables as potential confounders a priori: lifestyle habits (BMI, cigarette smoking, alcohol consumption, and time spent walking in hours per day), family history of cancer, past history of diseases, and socioeconomic factors (marital status and education). We also examined effect modification by age, sex, and other covariates.

All statistical analyses were performed using SAS software, version 9.1. All the statistical tests reported were two-sided; a P value of <0.05 was considered statistically significant.

## RESULTS

The mean scores (SD) on EPQ-R subscales for our participants were 5.64 (3.13) for extraversion, 5.43 (3.06) for neuroticism, 3.34 (1.67) for psychoticism, and 7.27 (2.57) for lie. These values are consistent with those reported for the general population.^17^

### Characteristics of participants in the highest and lowest score categories of the 4 personality subscales

We compared the characteristics of participants in the highest and lowest categories (ie, approximate quartiles) of each personality subscale (Table 1). As compared to participants in the lowest category, subjects in the highest category of extraversion were more likely to be male, to be overweight, to walk, to be a current smoker, and to be a current drinker. Participants in the highest category of neuroticism were more likely to have a history of disease, and were less likely to be male, to be overweight, and to walk. Those in the highest category of psychoticism were more likely to be male, to be a current smoker, and to be a current drinker. Participants in the highest category of lie were more likely to have a history of disease, to walk, and to have a low education level (in school until age ≤15), and less likely to be male, to be a current smoker, and to be a current drinker.

**Table 1.** Characteristics of participants according to highest and lowest categories of scores on 4 personality subscales (n = 21,911)

	Extraversion		Neuroticism		Psychoticism		Lie	
	
Personality subscale^*^ (score)	≤3	≥9	≤3	≥8	≤2	≥5	≤5	≥10
Number of participants	6322	4712	6635	5965	7308	4955	5206	4493
Mean age, years (SD^†^)	51 (8)	51 (7)	51 (8)	51 (8)	52 (8)	50 (7)	48 (7)	54 (7)
Men (%)	42	49	49	42	31	66	57	40
Body mass index, kg/m^2^ (%)								
<18.5	3	2	1	3	2	2	2	2
18.5-24.9	71	63	65	70	69	67	69	69
≥25.0	26	35	34	27	29	31	29	29
Past history of disease^‡^ (%)	31	31	28	36	33	30	29	34
Family history of cancer (%)	28	28	28	28	29	26	27	28
Time spent walking in hour/day (%)								
≥1	41	47	46	42	44	42	40	49
0.5-1	25	25	26	24	26	24	26	24
≤0.5	34	28	28	35	30	34	34	27
Alcohol (%)								
Current drinker	45	61	54	52	43	64	63	44
Ex-drinker	5	4	4	5	4	6	5	4
Never drinker	50	35	42	43	53	30	33	52
Cigarette smoking (%)								
Current smoker	29	38	36	31	22	49	42	28
Ex-smoker	11	11	10	12	9	13	12	10
Never smoker	60	51	54	58	69	38	45	63
Marital status (%)								
Married	89	91	91	89	88	91	91	89
Separated/divorceed/widowed	7	6	6	7	8	5	5	8
Single	4	2	3	3	3	4	4	3
Education, years of age (%)								
<16	37	33	35	36	34	36	29	41
16-18	48	50	49	50	51	48	53	46
≥19	15	17	17	15	15	16	18	14

^*^Each personality subscale (scored on a scale of 0-12) was divided into 4 categories of approximately equal size on the basis of the score for the population. Consequently, different personality subscales have different cut-off scores.

^†^Standard deviation.

^‡^Past history of stroke, hypertension, myocardiac infarction, renal diseases, liver diseases, gallstone diseases, diabetes mellitus, peptic ulcers, or tuberculosis.

### Personality subscales and gastric cancer screening compliance

Table 2 compares the association between personality and gastric cancer screening compliance (attending every year vs. other). There was a statistically significant linear, positive association between extraversion score and gastric cancer screening compliance (*P* < 0.001 for trend); a higher score was associated with increased odds of attending gastric cancer screening every year (compliance). There was a statistically significant linear, inverse association between neuroticism score and gastric cancer screening compliance (*P* = 0.047 for trend); a higher score was associated with decreased odds of gastric cancer screening compliance. There was a statistically significant linear, inverse association between psychoticism score and gastric cancer screening compliance (*P* < 0.001 for trend); a higher score was associated with decreased odds of gastric cancer screening compliance. The lie score was not associated with gastric cancer screening compliance (*P* = 0.32 for trend).

**Table 2.** Results of logistic regression analysis of personality and gastric cancer screening compliance (n = 21,911)

	Category^*^				Trend *P*-value*^†^*
	
Personality subscale	1 (reference)	2	3	4	
Extraversion	≤3	4 - 5	6 - 8	≥9	
No. of screened patients/no. of participants	1571/6322	1310/4800	1726/6077	1386/4712	
Age- and sex-adjusted OR (95% CI)	1.00	1.10 (1.01 - 1.21)	1.15 (1.06 - 1.25)	1.22 (1.12 - 1.33)	<0.001
Multivariate adjusted OR (95% CI)^‡^	1.00	1.10 (1.01 - 1.20)	1.16 (1.07 - 1.26)	1.21 (1.11 - 1.32)	<0.001

Neuroticism	≤3	4 - 5	6 - 7	≥8	
No. of screened patients/no. of participants	1921/6635	1303/4788	1219/4523	1550/5965	
Age- and sex-adjusted OR (95% CI)	1.00	0.93 (0.86 - 1.02)	0.94 (0.86 - 1.02)	0.91 (0.84 - 0.98)	0.015
Multivariate adjusted OR (95% CI)^‡^	1.00	0.94 (0.87 - 1.03)	0.95 (0.87 - 1.04)	0.92 (0.85 - 1.00)	0.047

Psychoticism	≤2	3	4	≥5	
No. of screened patients/no. of participants	2133/7308	1487/5368	1147/4280	1226/4955	
Age- and sex-adjusted OR (95% CI)	1.00	0.92 (0.85 - 0.99)	0.89 (0.81 - 0.97)	0.79 (0.73 - 0.87)	<0.001
Multivariate adjusted OR (95% CI)^‡^	1.00	0.93 (0.86 - 1.01)	0.91 (0.83 - 0.99)	0.84 (0.77 - 0.92)	<0.001

Lie	≤5	6 - 7	8 - 9	≥10	
No. of screened patients/no. of participants	1284/5206	1432/5543	1871/6669	1406/4493	
Age- and sex-adjusted OR (95% CI)	1.00	0.97 (0.89 - 1.06)	0.99 (0.90 - 1.08)	1.02 (0.92 - 1.12)	0.49
Multivariate adjusted OR (95% CI)^‡^	1.00	0.98 (0.90 - 1.07)	1.00 (0.91 - 1.09)	1.03 (0.94 - 1.14)	0.32

^*^Each personality subscale (scored on a scale of 0-12) was divided into 4 categories of approximately equal size on the basis of the score for the population. Consequently, different personality subscales have different cut-off scores.

^†^Trend *P*-value was calculated by treating personality subscales as continuous variables.

^‡^Adjusted for age, sex, lifestyle variables including cigarette smoking (current smokers, ex-smokers, or never smokers), alcohol drinking (current drinkers, ex-drinkers, or never drinkers), body mass index (<18.5, 18.5-24.9, or ≥25.0), and hours of walking per day (more than 1 hour, 0.5-1 hour, or less than 0.5 hours), family history of cancer (presence or absence), past histories including stroke, hypertension, myocardial infarction, renal diseases, liver diseases, gallstone diseases, diabetes mellitus, and tuberculosis (presence or absence), and socioeconomic variables including education level (in school until age 15 years or less, 16-18 years, 19 years or high), marital status (married, separated/divorced/widowed, or single).

All odds ratios (ORs) are shown with 95% confidence intervals (CIs) in parentheses.

### Personality subscales and gastric cancer screening visiting

Table 3 compares the association between personality and gastric cancer screening visiting (attending ≥1 time in 5 years vs. never attending).

**Table 3.** Results of logistic regression analysis of personality and gastric cancer screening visiting (n = 21,911)

	Category^*^				Trend *P*-value^†^
	
Personality subscale	1 (references)	2	3	4	
Extraversion	≤3	4 - 5	6 - 8	≥9	
No. of screened patients/no. of participants	4057/6322	3216/4800	4112/6077	3215/4712	
Age- and sex-adjusted OR (95% CI)	1.00	1.11 (1.03 - 1.20)	1.13 (1.05 - 1.22)	1.16 (1.07 - 1.26)	<0.001
Multivariate adjusted OR (95% CI)^‡^	1.00	1.11 (1.02 - 1.20)	1.14 (1.05 - 1.23)	1.16 (1.07 - 1.26)	<0.001

Neuroticism	≤2	3 - 4	5 - 7	≥8	
No. of screened patients/no. of participants	4438/6635	3182/4788	2988/4523	3992/5965	
Age- and sex-adjusted OR (95% CI)	1.00	0.99 (0.92 - 1.08)	0.99 (0.91 - 1.07)	1.05 (0.97 - 1.13)	0.23
Multivariate adjusted OR (95% CI)^‡^	1.00	1.00 (0.92 - 1.08)	0.99 (0.92 - 1.08)	1.05 (0.97 - 1.13)	0.21

Psychoticism	≤2	3	4	≥5	
No. of screened patients/no. of participants	5064/7308	3590/5368	2862/4280	3084/4955	
Age- and sex-adjusted OR (95% CI)	1.00	0.89 (0.83 - 0.96)	0.91 (0.84 - 0.99)	0.74 (0.69 - 0.81)	<0.001
Multivariate adjusted OR (95% CI)^‡^	1.00	0.90 (0.83 - 0.97)	0.92 (0.85 - 1.00)	0.78 (0.72 - 0.84)	<0.001

Lie	≤4	5 - 6	7 - 8	≥9	
No. of screened patients/no. of participants	3278/5206	3661/5543	4513/6669	3148/4493	
Age- and sex-adjusted OR (95% CI)	1.00	1.06 (0.98 - 1.15)	1.05 (0.97 - 1.13)	1.05 (0.96 - 1.15)	0.14
Multivariate adjusted OR (95% CI)^‡^	1.00	1.07 (0.99 - 1.17)	1.07 (0.98 - 1.15)	1.07 (0.98 - 1.18)	0.053

^*^Each personality subscale (scored on a scale of 0-12) was divided into 4 categories of approximately equal size on the basis of the score for the population. Consequently, different personality subscales have different cut-off scores.

^†^Trend *P*-value was calculated by treating personality subscales as continuous variables.

^‡^Adjusted for age, sex, lifestyle variables including cigarette smoking (current smokers, ex-smokers, or never smokers), alcohol drinking (current drinkers, ex-drinkers, or never drinkers), body mass index (<18.5, 18.5-24.9, or ≥25.0), and hours of walking per day (more than 1 hour, 0.5-1 hour, or less than 0.5 hours), family history of cancer (presence or absence), past histories including stroke, hypertension, myocardial infarction, renal diseases, liver diseases, gallstone diseases, diabetes mellitus, and tuberculosis (presence or absence), and socioeconomic variables including education level (in school until age 15 years or less, 16-18 years, 19 years or high), marital status (married, separated/divorced/widowed, or single).

All odds ratios (ORs) are shown with 95% confidence intervals (CIs) in parentheses.

There was a statistically significant linear, positive association between extraversion score and gastric cancer screening visiting (*P* < 0.001 for trend); a higher score was associated with increased odds of attending gastric cancer screening 1 or more years (visiting). The neuroticism score was not associated with gastric cancer screening visiting (*P* = 0.21 for trend). There was a statistically significant linear, inverse association between psychoticism score and gastric cancer screening visiting (*P* < 0.001 for trend); a higher score was associated with decreased odds of gastric cancer screening visiting. The lie score was not associated with gastric cancer screening visiting (*P* = 0.053 for trend).

We repeated the same analysis after adding the 4521 participants who reported having peptic ulcers with the study participants in the original analysis; the results did not change regarding extraversion (*P* < 0.001 for trend) and psychoticism (*P* < 0.001 for trend). A similar result was observed with neuroticism, although it was not statistically significant (*P* = 0.079 for trend). On the lie subscale, there was a statistically significant linear, positive association between the lie score and gastric cancer screening compliance (*P* = 0.032 for trend). We also examined the effects of modification by age, sex, lifestyle habits, family history of cancer, history of diseases, and socioeconomic factors, on gastric cancer screening compliance and gastric cancer screening visiting. There was no interaction in all covariates (data not shown).

## DISCUSSION

In this population-based, cross-sectional study in a rural Japanese community, we found significant associations between scores on personality subscales and attendance at gastric cancer screening. There was a statistically significant linear, positive association between extraversion and gastric cancer screening attendance. There were also statistically significant linear, inverse associations between both psychoticism and neuroticism and gastric cancer screening attendance.

Five earlier studies investigated associations between psychological state and cancer screening attendance.^11^^–^^15^ Two studies focused on the association between neuroticism or extraversion and cancer screening visiting (attendance 1 or more times vs. no attendance). One study reported that neuroticism was not significantly associated with breast cancer screening visiting,^13^ which is consistent with our results. However, we found that neuroticism score was significantly associated with gastric cancer screening compliance. Thus, these results suggest that neuroticism score is more closely associated with cancer screening compliance than with cancer screening visiting.

One study reported that the association between extraversion and breast cancer screening visiting was no longer significant after adjusting for a variety of covariates.^14^ In our study, however, the statistically significant linear, positive association between extraversion score and gastric cancer screening visiting remained even after multivariate adjustment.

No previous study investigated the association between psychoticism or lie and cancer screening attendance. We found a statistically significant linear, inverse association between psychoticism score and both gastric cancer screening visiting and compliance; however, the lie score was not associated with either.

Our study had some methodological advantages over previous studies. First, we tested the association between personality and compliance with cancer screening. In Japan, it is recommended that people aged over 40 should attend gastric cancer screening once a year.^5^ Thus, in this study, compliance was defined as attending gastric cancer screening every year for the previous 5 years. In our examination of the association between personality subscales and the 2 definitions of gastric cancer screening attendance—compliance and visiting—the association with personality subscales was stronger with compliance than with visiting. For example, the neuroticism score was associated with gastric cancer screening compliance but not with visiting. Three of 5 earlier studies examined the association between psychological state and cancer screening visiting, which might partially account for the inconsistent/negative results of the 3 studies. On the other hand, we noted a significant linear association between 3 personality subscales—extraversion, neuroticism, and psychoticism—and cancer screening compliance.

Second, to examine the effect of each confounder on personality, we considered the variables of lifestyle habits, family history of cancer, history of diseases, and socioeconomic factors. However, the results did not change when these variables were considered (Tables 2 and 3).

Several methodological limitations, however, should be considered when interpreting our results. First, our study had a cross-sectional design; thus, we observed only a temporal relationship between personality and gastric cancer screening attendance. Second, the number of analyzed participants was 21,911, which was 53% of all participants in the study. We excluded participants with unreliable/missing responses to the EPQ-R (n = 8654), a past history of cancer (n = 730), questionnaires that had been completed with the aid of family members (n = 2437), a past history of peptic ulcers (n = 4521), and incomplete responses regarding gastric cancer screening attendance (n = 3171). The mean age of participants who were included in the study (21,911 participants) was slightly lower than that of those who were excluded (19,513 participants) (51 vs. 53). However, the sex ratio and lifestyle habits of participants who were included in the study and those who were excluded were similar (male, 45% vs. 50%; overweight, 30% vs. 27%; history of disease, 32% vs. 35%; current smoker, 30% vs. 34%; current drinker, 48% vs. 46%, respectively). Third, we used a self-reported questionnaire to determine the frequency of gastric cancer screening attendance in the previous 5 years; participants answers were subject to mistakes in recollection and other errors. Fourth, the present study examined the relation between personality and gastric cancer screening attendance only. It is uncertain whether the present findings apply to attendance at screenings for other types of cancer. The present findings should be re-evaluated in different populations and for different types of cancer.

We believe that a better understanding of the association between personality and screening attendance may lead to the establishment of an effective campaign to promote attendance at cancer screening programs. Based on the present findings, we offer the following 2 hypotheses on which to base strategies for achieving higher rates of participation in cancer screening.

First, there was a significant inverse association between neuroticism score and gastric cancer screening compliance, but not between neuroticism score and gastric cancer screening visiting. This indicates that individuals with higher neuroticism scores tend to undergo gastric cancer screening at least once, but not every year. Individuals who do not regularly attend cancer screening are more likely to have higher neuroticism scores than those who do so regularly. Accordingly, compliance among these participants might be improved by taking into account the neurotic aspects of their personality by, for instance, providing emotional support to relieve any concerns about screening results, emphasizing the benefit to be gained from a screening program, and minimizing any anxiety or discomfort during the test.

Second, there was a significant inverse association between psychoticism score and gastric cancer screening compliance and visiting. These results suggest that individuals with higher psychoticism scores tend not to undergo gastric cancer screening at all. In other words, those who do not attend for cancer screening are more likely to have higher psychoticism scores. Accordingly, such individuals might be encouraged to undergo cancer screening by taking into account the psychotic aspects of their personality. It is well known that individuals with higher psychoticism scores tend to decide and act by themselves. Thus, a phrase emphasizing autonomy or independence, such as “Take positive steps to protect yourself by having a health exam”, might provide more effective encouragement.

These two hypotheses have been developed solely on the basis of the present results and have not yet been tested. However, as the rate of participation in cancer screening programs in Japan is still lower than in Europe and the United States, we need to step up our ongoing campaign and devise new strategies to improve attendance at cancer screenings. Here, we have proposed new hypotheses from the perspective of personality. We hope that hypotheses will be tested by intervention trials.

In conclusion, our data support the hypothesis that aspects of personality, such as extraversion, neuroticism, and psychoticism, influence attendance at gastric cancer screenings. It is clear that personality has a major influence on health-related behavior and disease risk, but the mechanisms and interactions involved have not been adequately clarified. In order to establish more effective comprehensive strategies for health promotion and disease prevention, the association between personality and health should be further investigated.
